# Offering e‐cigarettes for smoking cessation and reduction in people with mental illness (ESCAPE): Protocol for a randomized controlled trial

**DOI:** 10.1111/add.70115

**Published:** 2025-07-01

**Authors:** Dimitra Kale, Emma Beard, Yan Ding, Jodi Pervin, Qi Wu, Catherine Arundel, Steve Parrott, Paul Galdas, Michelle Horspool, Simon Hough, Gregor Russell, Suzy Ker, Elena Ratschen, Lion Shahab

**Affiliations:** ^1^ Department of Behavioural Science and Health University College London London UK; ^2^ Department of Health Sciences University of York York UK; ^3^ Sheffield Health and Social Care NHS Foundation Trust Sheffield UK; ^4^ Representative of Patient and Public Involvement Group; ^5^ Bradford District Care NHS Foundation Trust Bradford UK; ^6^ Tees, Esk and Wear Valleys NHS Foundation Trust Darlington UK

**Keywords:** E‐cigarette, harm reduction, mental illness, protocol, randomized controlled trial, smoking cessation, vaping

## Abstract

**Background and aims:**

Despite a steady decline in smoking rates across the United Kingdom (UK) over the past decades, substantial tobacco‐related inequalities persist, particularly among individuals with mental illness. Smoking prevalence in this group has remained largely unchanged, highlighting a major public health concern. This protocol outlines a trial aimed at addressing this issue by evaluating the effectiveness of offering an electronic cigarette (e‐cigarette) starter kit for smoking cessation and harm reduction as an adjunct to usual care to adults who smoke with mental illness treated in the community.

**Design:**

Two‐arm parallel randomized controlled superiority trial.

**Setting:**

A minimum of eleven UK National Health Service mental health Trusts or general practices.

**Participants:**

Adults who smoke with a diagnosis of mental illness treated in the community.

**Intervention and comparator:**

Participants will be randomly allocated (1 : 1) to receive: (i) an e‐cigarette starter kit, a brief demonstration and both verbal and written information on e‐cigarette use as an adjunct to usual care; or (ii) usual care alone.

**Measurements:**

The primary outcome is 7‐day point prevalence carbon monoxide‐validated abstinence, assessed six months after study enrolment or target quit date. Secondary outcomes include: 6‐month continuous abstinence defined by Russell Standard, self‐reported smoking abstinence at 1 month, ≥50% smoking reduction (cigarettes/day) at 1 and 6 months, mental health symptoms and general mood and physical symptoms at 1 and 6 months, cost‐effectiveness of the intervention and adherence to and satisfaction with the intervention.

**Comments:**

This will be the first large‐scale study in the UK to assess the effectiveness and cost‐effectiveness of providing an e‐cigarette starter kit for smoking cessation and harm reduction in people with mental illness. Results will inform policy and practice related to supporting smoking cessation for this population.

## INTRODUCTION

Tobacco smoking accounted for 15% of deaths in the UK general population in 2019 [1]. Tobacco‐related deaths and diseases are not evenly distributed across the population, with higher rates of tobacco‐related morbidities among individuals with mental health illness [2]. People with mental health illness are more than twice as likely to smoke compared with the general population, and there has been limited change in smoking prevalence among this group over the past five decades [2, 3, 4]. Despite being similarly motivated [5] and able to [6] quit smoking as those without mental illness, they face greater challenges owing to high nicotine dependence and limited access to support, including a reduced likelihood of prescribers offering stop‐smoking medication and a reduced likelihood of clinicians offering to refer them to stop‐smoking services [7]. Therefore, it is crucial to develop more effective and tailored smoking cessation strategies for this population.

Electronic cigarettes (e‐cigarettes), which provide nicotine without many of the harmful substances found in tobacco smoke, are increasingly recognized as a valuable tool for smoking cessation for people with mental illness [8]. In England, there has been an increase in their use as a smoking cessation tool [9, 10], with nicotine‐containing e‐cigarettes being included as a treatment component in national guidelines on the treatment of tobacco dependence [11]. In the general population, accumulating evidence indicates that nicotine e‐cigarettes are more effective than nicotine replacement therapy (NRT) in aiding smoking cessation [12, 13, 14]. Additionally, research conducted in Italy [15, 16], the USA [17], Australia [18] and the UK [19] suggests that e‐cigarettes can be effective for smoking cessation and reduction among adults with mental illness who smoke. While cessation remains the ideal outcome, smoking reduction also highlights the potential to reduce morbidity and mortality associated with smoking, as even partial reductions in cigarette consumption can yield health benefits [20]. Importantly, these studies did not find any evidence that e‐cigarettes adversely affect mental health, further emphasising their potential as a safe smoking cessation aid for individuals with mental illness. In fact, the uptake of e‐cigarettes appears particularly high among people with mental illness, who have been shown to be more likely to have tried and to currently use e‐cigarettes than the general population [21]. Several factors may contribute to this. First, e‐cigarettes are relatively inexpensive compared with both cigarettes and other cessation treatments [22]. Second, they offer a straightforward stand‐alone treatment that is easy to use. Third, unlike most NRTs, e‐cigarettes mimic the sensory experience of smoking and allow users to control their nicotine intake, which may appeal to people who are more dependent upon smoking and have previously struggled to quit [23]. As such, e‐cigarettes could be an important aid in efforts to address tobacco‐related health inequalities in this population, which has been identified as a health priority in England [24, 25]. As service models to improve the provision of tobacco dependence treatment are being rolled out across the country [25], it is important to advance the evidence base on the role that e‐cigarettes can play in these efforts.

To explore the feasibility and acceptability of supplying e‐cigarette starter kits, along with brief verbal and written advice on e‐cigarette use, as an adjunct to usual care for smoking cessation in adults who smoke with mental illness treated in the community, we conducted a feasibility study [26]. The results demonstrated that the trial and intervention were acceptable to both participants and health professionals delivering the treatment [26]. Although the target size was not achieved, the feasibility study obtained good consenting and adherence rates with limited contamination. Additionally, an exploratory analysis suggested trends favouring the intervention for continuous abstinence [for weeks 2–4 from the enrolment date or the target quit date (for those who set a date within 1 week of enrolment)], point prevalence abstinence (24‐hour abstinence) and ≥50% reductions in cigarette consumption [26, 27, 28]. The cost of the intervention was comparable with similar smoking cessation treatments for this population. Our feasibility study also demonstrated the feasibility of collecting data on healthcare resource use and quality of life among people with mental illness [26], which is essential for conducting a comprehensive health economic evaluation.

We used learning from the feasibility study to optimize the methodology for conducting a subsequent definitive RCT to evaluate the effectiveness and cost‐effectiveness of offering an e‐cigarette starter kit as an adjunct to usual care for smoking cessation and reduction in people with mental illness (ESCAPE), the protocol for which is presented here.

### Objectives

#### Primary

To assess the impact of offering an e‐cigarette starter kit intervention, compared with usual care alone, on 7‐day point prevalence carbon monoxide (CO)‐validated abstinence from smoking at the 6‐month follow‐up.

#### Secondary

1. To evaluate the impact of offering an e‐cigarette starter kit intervention, compared with usual care alone, on:
  - self‐reported continuous smoking abstinence at the 6‐month follow‐up;
  - self‐reported smoking cessation rates at the 1‐month follow‐up;
  - self‐reported smoking reduction at both the 1‐ and 6‐month follow‐ups;
  - mental health outcomes and psychosocial measures at the 6‐month follow‐up.
2. To assess the cost‐effectiveness of offering the e‐cigarette starter kit intervention over and above usual care.
3. To assess the adherence to and satisfaction with the e‐cigarette starter kit intervention.

## METHODS

### Design and setting

This is a two‐arm parallel randomized controlled superiority trial to assess the effectiveness of offering an e‐cigarette starter kit intervention, compared with usual care, for increasing abstinence rates at the 6‐month follow‐up (Data S1). The study will take place in a minimum of 11 primary and secondary care UK National Health Service (NHS) mental health trusts and general practices (GPs). Although specialist mental health services in England are mostly provided by mental health trusts, classified as secondary care, most patients with mental illness, particularly with common conditions such as anxiety and depression, are managed in primary care. Clinical contact with patients with mental illness in primary care is likely to be in the form of medication/mental health reviews, physical health checks or access to low‐intensity therapies. Patients managed in secondary care services are more likely to have severe, complex and enduring mental illness, such as schizophrenia and bipolar disorder, and receive treatment and care from specialist mental health professionals in multidisciplinary teams.

### Participants

Participants will be adults who smoke, have a diagnosis of mental illness based on their medical records, and meet all of the inclusion criteria and none of the exclusion criteria.

### Inclusion criteria

Participants will be included if they: (i) are aged ≥18 years; (ii) are self‐reported current cigarette smokers (in the past 7 days); (iii) have a diagnosis of mental illness (in accordance with UK NHS categorization, https://www.nhs.uk/mental-health/conditions/) and are currently being managed or receiving treatment for this in primary or secondary care, validated by their healthcare records; (iv) have the capacity to provide consent; and (v) are seriously considering quitting, cutting down or maintaining abstinence from tobacco.

### Exclusion criteria

Participants will be excluded if they: (i) have had a mental health inpatient admission in the last 3 months; (ii) have self‐reported current regular use of e‐cigarettes (at least weekly); (iii) have self‐reported current participation in another smoking cessation trial; (iv) are receiving current treatment for comorbid drug or alcohol problems to reduce the possibility of hospital admission for acute events (and thus the risk of dropout); (v) have a diagnosis of Alzheimer's disease or dementia; (vi) have a nut, citrus, nicotine or propylene glycol allergy; or (vii) are pregnant or breastfeeding.

### Treatment groups

#### Control

Participants in the control group will receive usual care related to smoking cessation in accordance with UK National Institute for Health and Care Excellence (NICE) guidelines. Usual care, which is free to access in the UK, varies between trusts and GP practices but is guided by NICE guideline NG209 [11]. At a minimum, it should include evidence‐based and very brief advice to stop smoking, which consists of the three As (Ask and record smoking status; Advice on the best way of quitting; and Act on patient response to build confidence) [29, 30], and a referral to in‐house or external specialist stop‐smoking services. Participants, regardless of their motivation to stop smoking, will be encouraged to consider quitting and to set a target quit date within a week of randomization. Those who do not wish to set a target quit date will be encouraged to reduce their cigarette consumption.

#### Intervention

Participants allocated to the intervention group will be offered an e‐cigarette starter kit, and a verbal explanation and demonstration, along with an information leaflet on how to use the e‐cigarette as an adjunct to the usual care outlined above. The e‐cigarette starter kit will contain a pod‐based e‐cigarette (ICON Vape), which is suction‐automated and uses pre‐filled 1.6‐mL pods containing 20 mg/mL nicotine e‐liquid. We chose a high nicotine concentration based on pervious work in this population, because smokers with mental illness have higher dependence [31] and e‐cigarettes with higher nicotine concentrations tend to be more effective [16]. All intervention participants will be provided with a 4‐week supply of refill pods (seven boxes) in tobacco, menthol and fruit flavours in a 1 : 2 : 2 ratio, based on the choice of flavour preferences observed during the feasibility study. All intervention participants will be able to choose the flavour of the final two boxes. The ICON Vape device was selected based on consumer research undertaken by the University of East Anglia [32, 33, 34], where it received high ratings for ease of use, satisfaction, taste and flavour options. All intervention participants, regardless of their motivation to stop smoking, will be encouraged to consider quitting and to set a target quit date within a week of randomization. They will be asked to start using the e‐cigarette as soon as possible and to seek out local or online vape shops to obtain further e‐liquid pods, suited to their individual needs and flavour preference. Participants who do not wish to set a target quit date will be encouraged to use the e‐cigarette to reduce cigarette consumption as soon as possible.

### Sample size

We aim to recruit 616 participants, with 308 participants asigned to each group. Pooling the results of the only three RCTs comparing e‐cigarettes with placebo e‐cigarettes [15, 35, 36] yielded an expected effect size of risk ratio (RR) = 2.01 for 6‐month abstinence rates. We consider this estimate to be conservative as most of these trials used older, less effective first‐generation e‐cigarettes. In fact, recently published trials comparing active treatment (NRT) with a modern third‐generation e‐cigarette in the context of stop‐smoking services [12] found a superior effect of e‐cigarettes on 6‐month abstinence rates of RR = 1.63. Based on established effect estimates for NRT versus placebo from high‐quality trials [37], findings from this trial therefore indirectly suggest that, compared with placebo/usual care, third‐generation e‐cigarettes (as will be used in this trial) may increase 6‐month abstinence rates by nearly threefold (RR = 2.62). Given the low abstinence rates in the control conditions observed in the feasibility study [26], we revised the expected abstinence rates in the control (standard care) group from 7.7% (based on previous trials with people with mental illness) [38, 39] to 5.0%. Based on the feasibility study results (finding an absolute risk difference of 9.5%) [26] and the fact that a modern e‐cigarette is used, we assume an effect estimate of RR = 2.62, as found in more recent trials, to be most appropriate. We therefore expect an absolute predicted risk difference of 8.1%, assuming 6‐month abstinence rates of 5% in the control group and 13.1% in the intervention (e‐cigarette) group. A sample of 616 participants (308 per group) would provide more than 90% power to detect this effect, with an alpha level of 0.05 in two‐tailed analysis. This sample size would also provide 80% power to detect a more conservative effect of RR = 2.32 (intermediary between effect estimates from older and effect estimates from more recent trials using modern e‐cigarettes), with an absolute predicted risk difference of 6.6% at the 6‐month follow‐up (5.0% for the control group vs 11.6% for the treatment group).

No adjustment for attrition is applied to the sample size calculations, as participants lost to follow‐up will be classified as people who smoke, as defined by the Russell Standard [40]. There is also no inflation using a design effect to account for clustering in trusts as it is assumed that the intra‐cluster correlation is likely to be very small or negligible [41].

### Procedure

**FIGURE 1:**
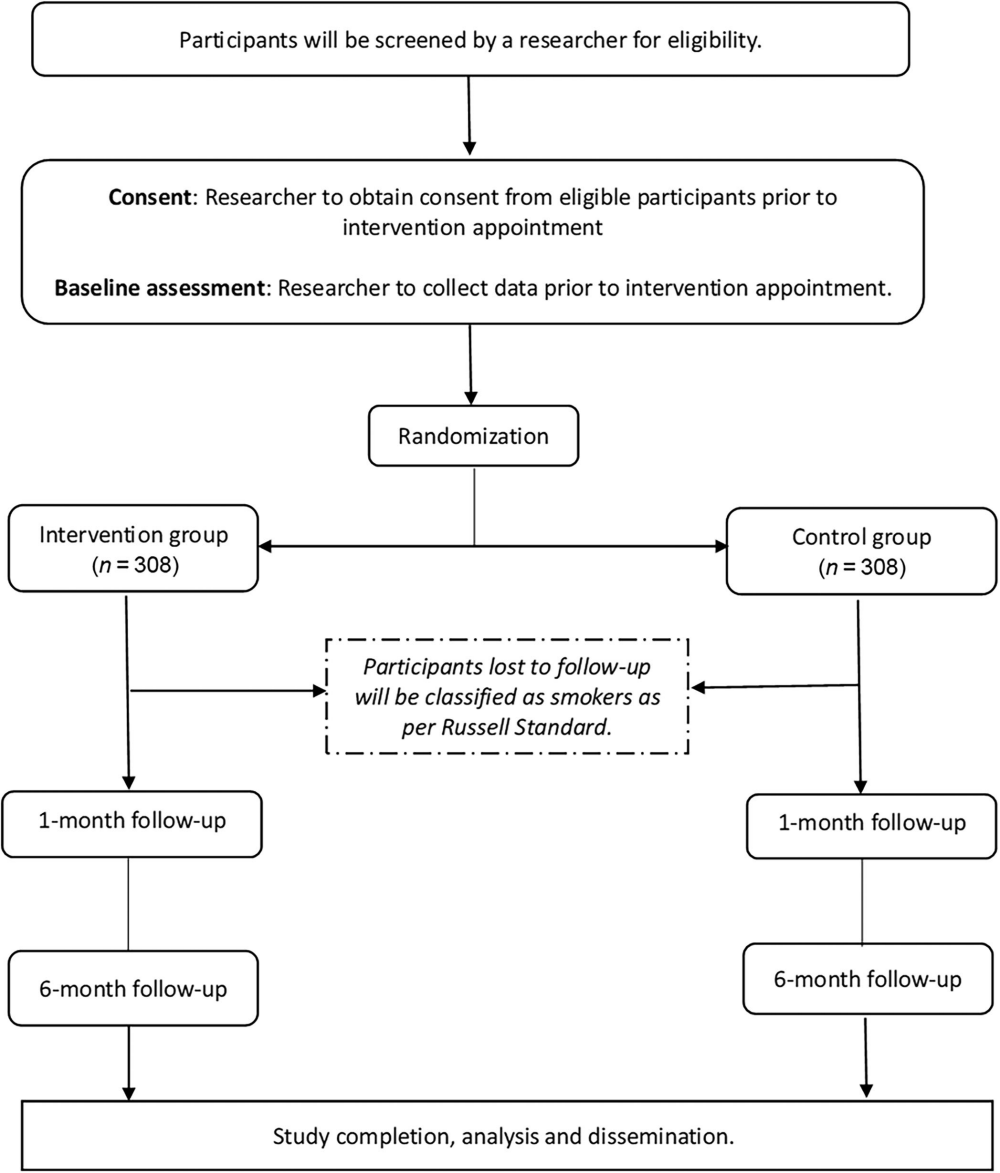
Study flow chart.

Figure 1 presents the study flow diagram and Table 1 presents the schedule of assessment at baseline and at the follow‐ups.

**TABLE 1:** Schedule of enrolment, interventions and assessments.

Activities	Study period			
	Identification	Allocation	Follow‐up	
	Baseline	0	1 month	6 months
**Enrolment**				
Eligibility screen	x			
Informed consent	x			
Allocation		x		
**Interventions**				
Usual care		x		
E‐cigarette starter kit, brief demonstration, verbal and written information on e‐cigarette use as an adjunct to usual care		x		
**Assessments**				
Socio‐demographic characteristics	x			
Nicotine dependence	x			
Motivation to quit	x			
Age started smoking	x			
Smoking duration	x			
Past year quit attempts	x			
Current use of tobacco products other than cigarettes	x			
Ever e‐cigarette use as smoking cessation aid	x			
AUDIT‐C	x			x
Physical activity	x			x
Fruit and vegetable consumption	x			x
Patient Heath Questionnaire‐9	x		x	x
Generalized Anxiety Disorder Scale‐7	x		x	x
7‐day point prevalence			x	x
Sustained abstinence				x
50% smoking reduction			x	x
CO breath sample				x
General mood and physical symptoms			x	x
Adverse events			x	x
Use of e‐cigarette			x	x
Healthcare service use	x		x	x
Health‐related quality of life	x			x

Socio‐demographic characteristics include age, sex, employment status, ethnicity, education, accommodation type and marital status.

#### Recruitment

Recruitment commenced in March 2024 and is planned to continue until the end of 2024. Potential participants will be identified through a range of routes depending on trust/GP practice preference, including identification via health record search, clinic attendance lists or through an opportunistic approach. Participants will receive an invitation letter, a short infographic participant information sheet and a reply form with a stamped addressed envelope (if appropriate). As interest and motivation to participate in smoking cessation studies can vary [42], those who initially decline will be offered participation up to three times, through a letter, follow‐up call and, if possible, a text message. Participants who are interested will be asked to respond to indicate this verbally over the telephone, by returning a reply slip to the research team or by using an online link to register their interest.

#### Eligibility assessment

Participants who express an interest to participate in the study will receive a telephone call from a trust/GP‐based researcher to check eligibility using a formal screening checklist and to answer any questions that participants may have about the study.

#### Consenting and baseline assessment

After verifying eligibility, participants will be invited to meet with a trust/GP‐based researcher before their scheduled clinical appointment to complete their informed consent form and baseline questionnaire in person.

#### Randomization

A computer block‐randomization method (with blocks of four and six), managed by one of the lead researchers not involved in the identification and recruitment of participants, will be used to ensure that each trial site has an equal number of intervention and control group participants. The randomization sequence will be concealed by using consecutively numbered opaque sealed envelopes that contain the allocation information. Randomization will only occur after obtaining full informed consent, completing the baseline questionnaire and the trust/GP‐based researcher completing a randomization form, which is attached to the sealed envelope. Trust/GP‐based researchers will then inform the participant and the person identified to deliver the intervention, if it is not the trust/GP‐based researcher themselves, of the allocation.

#### Blinding

Owing to the nature of the intervention and the study design, participants, researchers and clinical staff administering the intervention cannot be blinded. As the follow‐up questionnaires differ for intervention and control groups, the outcome assessment will only be blinded for questionnaires that participants complete online, rather than those completed over the phone. However, the study statistician will remain blinded to participant allocation.

#### Intervention delivery

The intervention will be delivered by a member of staff trained in delivering the intervention and should last between 5 and 10 minutes. Delivery will be performed by the trust/GP‐based researcher or by a healthcare professional as part of the scheduled clinical appointment at the site location. To increase the reach, there was a change in mode of delivery for the intervention arm in month 7, moving from adjunct appointments only to a combination of adjunct and special appointments to deliver the intervention.

#### Follow‐up data collection

Follow‐up data at 1 month and at 6 months will be obtained: online, via a link sent to the participant email address; via telephone, with support from a trust/GP‐based researcher; or via a paper‐based postal return, depending on the participant's preference. Given the alternative scenarios of intervention delivery outlined above and the potential for bias in follow‐up data collection, we stipulate that if the intervention is delivered by a trust/GP‐based researcher, then an alternative trust/GP‐based researcher will conduct the follow‐up data collection. As self‐reported abstinence at the 6‐month follow‐up will be biochemically validated using the PICO smokerlyzer (https://www.bedfont.com/products/smokerlyzer), this will require an in‐person visit either at the clinical base or in participants' homes to obtain a CO breath measurement reading to verify this.

### Measures

Table 1 presents the different measures assessed at different points throughout the trial.

#### Main outcomes

##### Primary outcomes measures

Self‐reported 7‐day point prevalence abstinence assessed 6 months after study enrolment or target quit date (for those who set a target quit date within 1 week of enrolment) and verified by exhaled breath CO < 10 ppm, which will be collected within 7 days of the 6‐month follow‐up assessment.

##### Secondary outcomes measures

Continuous abstinence at the 6‐month follow‐up, defined as not having had more than five cigarettes in the period from 2 weeks after baseline to the follow‐up, in line with the Russell Standard [40]. Self‐reported smoking abstinence at 2–4 weeks from baseline or target quit date (whichever is later). Fifty percent smoking reduction at 1‐ and 6‐month follow‐ups, calculated by the reduction in self‐reported cigarettes per day from baseline. Number of unsuccessful quit attempts at the 1‐ and 6‐month follow‐ups.

Mental health symptoms assessed by Patient Health Questionnaire‐9 (PHQ‐9) [43] and General Anxiety Disorder‐7 (GAD‐7) [44], general mood, physical symptoms assessed by the Mood and Physical Symptoms Scale (MPPS) [45, 46], and experience of adverse events at the 1‐ and 6‐month follow‐ups, similar to previous research [23].

Cost‐effectiveness of the intervention based on health economics measures outlined below.

The fidelity of intervention implementation and satisfaction with the intervention at the 1‐ and 6‐month follow‐ups will be based on questions related to e‐cigarette use in the past month as well as a question to assess whether the e‐cigarette used in the last month was the device provided to intervention participants as part of the study.

#### Baseline measures

We will record age, sex, employment status, ethnicity, education, accommodation type and marital status (Table 2), as well as most recent diagnosis and acute events (e.g. hospitalization) in the last year based on the participants' medical records. We will also assess nicotine dependence with the Strength of Urges to Smoke Scale [47] and motivation to quit with the Motivation to Stop Scale [48]. Age started smoking, smoking duration, past year quit attempts, current use of tobacco products other than cigarettes and ever e‐cigarette use as a smoking cessation aid will also be recorded. Alcohol use will be measured using the Alcohol use disorders identification test for consumption (AUDIT‐C) [49]. Physical activity (strength training; moderate or vigorous aerobic physical activity) will be assessed with questions from the Allied Dunbar National Fitness survey [50]. Consumption of at least five portions of fruit and vegetables (Healthy Eating Index) [51] will also be recorded.

**TABLE 2:** Measures of participant sociodemographic characteristics.

Characteristic	Question	Answer options
Age	What is your age?	18–99 years
Gender	What is your gender?	(i) Male (ii) Female (iii) Other
Ethnicity	How would you describe your ethnic background?	(i) White (ii) Mixed/Multiple (iii) Asian/Asian British (iv) Black/African/Caribbean/Black (v) British (vi) Chinese (vii) Arab (viii) Other
Education	What is your highest educational qualification?	(i) GCSE/O‐level/CSE (ii) Vocational qualification (iii) A‐level or equivalent (iv) Bachelor’s degree or equivalent (v) Master’s/PhD or equivalent (vi) No formal qualifications (vii) Other
Employment status	How would you describe your employment status?	(i) Employed full‐time (ii) Employed part‐time (iii) Self‐employed (iv) Retired (v) Student (vi) Voluntary worker (vii) Not employed but seeking work (viii) Not employed but not seeking work because of ill health (ix) Not employed but not seeking work for other reason (x) Other
Accommodation type	What is your current accommodation type?	(i) Purchasing with a mortgage (ii) Owned outright (iii) Rented from the local authority (iv) Rented from a private landlord (v) Rented from a housing association/trust (vi) Other
Marital status	What is your marital status?	(i) Single (ii) Married (iii) Living with partner/cohabiting (iv) Divorced/separated (v) Widowed (vi) Other

### Adverse events

At the 1‐ and 6‐month follow‐ups, participants will be asked to indicate if they have experienced any adverse events over the previous month and if these events stopped them from doing things they would normally do. These include the most common adverse events in previous trials, such as nausea, shortness of breath, throat/mouth irritation, increased appetite and headache [23].

### Health economics

A service use questionnaire (Table 3) piloted in the feasibility trial will be used to collect resource use at baseline and at the 1‐ and 6‐month follow‐ups, and will include both the cost of providing the interventions and the costs of patient healthcare utilization. Quality‐adjusted life years (QALYs) [52, 53] will be calculated using responses from the EuroQol five‐dimensional five‐level questionnaire (EQ‐5D‐5L) administered at baseline and at the 6‐month follow‐up.

**TABLE 3:** Service use questionnaire.

In the past month, how many times have you used the following health services that were NOT provided by the ESCAPE trial?		
	For smoking cessation/reduction	For any other purposes
	(Please enter the number of times you have used each service; if none, enter zero)	
Not in the hospital		
a. How many times have you seen your GP?	______times	______times
b. How many times have you seen a practice nurse?	______times	______times
c. How many times have you seen a pharmacist?	______times	______times
d. How many times have you attended a session with someone from a smoking cessation service?	______times	
e. How many times have you called the NHS Smoking Helpline service?	______times	
f. How many times have you seen a mental health worker in the community or at your home?	______times	______times
g. How many prescription items of the NRT product have you received?^a^	______ items	______ items
h. How many prescription items of Zyban (bupropion) have you received?	______ items	______ items
i. How many prescription items of Champix (varenicline) have you received?	______ items	______ items
j. How many prescription items have you received other than NRT, Zyban or Champix?	______ items	______ items
In the hospital		
k. How many times have you visited a hospital A&E department?		______times
l. How many times have you visited a hospital as an outpatient?		______times
m. How many times have you visited a hospital for a day case?^b^		______nights
n. How many nights have you stayed in a hospital as an inpatient?		______times
o. How many times have you been taken to the hospital by an emergency ambulance?		______times
p. None of the above	Yes	

^a^ One prescription item refers to a single prescribed medication, product or device on a prescription. If you have picked up the same medicine on several occasions over the period stated above, it counts as separate items for each occasion.

^b^ Day case: attended hospital for a procedure but stayed for less than 24 hours.

### Usual care and relevant contextual factors

Details and characteristics of the ‘usual care’ provided to the control group at each site will be collected at the end of the trial for assessment in the analysis. We will also document relevant contextual factors through the day‐to‐day and monthly scheduled communication between sites and the trial management and coordination team.

### Data management and monitoring

The data will be managed via the REDCap electronic data system [54, 55]. The research team based at University of York will enter the baseline data. Follow‐up data will be entered either by participants or by the research teams at the sites. Following data entry, data collected by paper questionnaire will be checked by another researcher based at University College London.

### Analysis

Data will be analysed in R Studio, with the alpha value set to 0.05. Outliers will be assessed and then handled based on clinically meaningful thresholds. This means only values that are unlikely to represent true clinical conditions are flagged. If outliers are retained in the analysis, sensitivity analysis will be performed to determine their impact on the results. Baseline characteristics will be reported by each arm using descriptive statistics: means and standard deviations (SDs) for continuous numeric variables [unless skewed, in which case the median and the interquartile range (IQR) will be reported instead]; and frequencies and percentages for categorical variables. Numeric variables include most recent diagnosis and acute events. All other variables are treated as categorical.

#### Primary analysis

The effect of treatment on the primary outcome (CO‐verified 7‐day abstinence rates at the 6‐month follow‐up) will be analysed by a mixed‐effect (multilevel) logistic regression model with fixed treatment and a random intercept for site, included to allow for any site‐level variation in treatment effect. We will employ a likelihood ratio (LR) test to assess the significance of including the random site effects by comparing the mixed‐effects model with a model including only fixed effects. In addition, we will calculate the intraclass correlation coefficient (ICC) to quantify the proportion of variance in the primary outcome attributable to differences among clinical sites.

Secondary outcomes at the 1‐ and 6‐month follow‐ups will also be analysed as described above if they are binary, or with mixed effects (multilevel) linear regression adjusting for site‐level variation if they are continuous numeric variables. Mean difference will be reported. For regressions, we will check the assumptions of normality of the residuals.

We will use a repeated measures analyses to examine changes in cigarette consumption (a reduction of ≥50%) and mental health symptoms across baseline, and the 1‐and 6‐month follow‐ups. For continuous mental health symptom scores, we will use a multilevel linear regression model. We will verify the assumptions of normality and homoscedasticity of the residuals using quantile–quantile (QQ) plots and residual versus fitted plots. If these assumptions are not met, we will apply appropriate transformations (e.g. log transformation for positively skewed data). For the binary outcome of a ≥50% reduction in cigarette consumption, we will use a mixed‐effects logistic regression model. We will assess for overdispersion by comparing the residual deviance with the degrees of freedom and will consider alternative models, such as negative binomial regression, if overdispersion is present. Our models will incorporate fixed effects for the intervention group, time (treated as a categorical factor) and their interaction to assess differential changes between groups over time. To account for individual variability, we will include random intercepts for each participant and clinic site.

Analyses of smoking‐related outcomes will follow the intention‐to‐treat principle. This means that all participants will be analysed in the groups to which they were originally assigned, regardless of whether they completed the study or adhered to the intervention. Following the Russell Standard [30], those participants who will be lost to follow‐up or who do not provide outcome data will be treated as people who smoke in the analysis. If the missing data mechanism is suspected to be missing at random (MAR), sensitivity analyses will be performed using multiple imputation and the last observation carried forward. MAR will be determined if significant differences are found between missing and observed data. If there are substantial changes in the results under different missingness assumptions this will also indicate MAR. A complete case and as‐treated analysis will also be conducted. The latter of which accounts for the fact that some in the control group may have used e‐cigarettes during the treatment period. In the event of a non‐significant difference between groups on primary and other outcomes, associated Bayes factors will be calculated using the method of Dienes to differentiate between evidence for no effect and data insensitivity [56], using the effect size from the sample size calculation as a prior and specifying a half‐normal distribution.

Two subgroup analyses will be conducted. The first to assess differences as a function of trust versus GP practice and the second to assess the impact of the change made in month 7 to the mode of delivery in the intervention arm. We do not pre‐specify subgroup analysis by severity of mental illness because of likely low recruitment of this population and associated limited power to detect effects.

There are challenges with achieving an appropriate sample size in studies recruiting vulnerable subpopulations. Thus, in a scenario where the sample size is not obtained, we plan a full Bayesian analysis. We will use Bayesian logistic regression to analyse the primary outcome of CO‐verified 7‐day abstinence rates at the 6‐month follow‐up. We will incorporate prior information from the feasibility study/sample size parameters into the model by using informative priors. Sensitivity analyses will be performed to assess the impact of different prior distributions. Instead of *P*‐values, we will focus on the posterior probability that the intervention is effective (e.g. the probability that the intervention reduces smoking rates) and report credible intervals.

### Economic evaluation

To determine the short‐term cost‐effectiveness of the intervention, we will undertake a within‐trial incremental cost‐effectiveness analysis to assess the value for money afforded by the intervention over and above usual care at 6 months, using data collected during the trial.

The resources used to provide the e‐cigarette starter kit intervention will be recorded alongside the trial. This includes staff time and materials used to train clinicians delivering the intervention, the provision of the e‐cigarette starter kit and the staff time spent on consultations. All participants will have access to usual care, and the use of currently available smoking cessation aids will be collected using a service use questionnaire piloted in the feasibility study at baseline, and at the 1‐ and 6‐month follow‐ups. Additionally, the participants' wider healthcare resource use in primary and secondary care will also be collected using the service use questionnaire. To generate cost profiles for each participant, unit costs derived from both the trial records and the most recent national average cost data will be applied to the quantities of corresponding resources used [57, 58]. QALYs will be calculated based on responses obtained from the EQ‐5D‐5L questionnaire using the ‘area under the curve’ approach, serving as the primary health outcome for the cost‐effectiveness analysis [52, 59]. We will assess patterns of missing data and assess the impact of missingness using multiple imputation methods [60].

A decision‐analytic model developed at the University of York will be adapted to project the long‐term cost‐effectiveness beyond the trial period [61]. Lifetime smoking‐attributable healthcare cost and expected QALYs associated with the two trial arms will be estimated using Markov models. The model will account for annual background abstinence for people who smoke and relapse for quitters, and an annual discount rate of 3.5% will be applied to both costs and QALYs [53, 62, 63]. For detailed methods, please refer to a previously published article [61].

Both within‐trial and model‐based economic evaluation will be conducted from an NHS and personal social services perspective, as recommended by NICE guidance [53]. Incremental cost‐effectiveness ratios (ICERs) will be calculated based on the mean differences in costs and QALYs estimated using regression methods [53, 64]. The ICERs will then be compared with the NICE recommended maximum acceptable ICERs of £20,000–30,000 per QALY to determine the probability that the intervention is cost‐effective at 6 months [53]. Sensitivity analyses will be conducted to test the robustness of the findings.

### Adherence to and satisfaction with the e‐cigarette starter kit intervention

The proportions of participants adhering to and satisfied with the intervention will be calculated. Adverse events will also be assessed descriptively.

### Data and safety monitoring

A trial steering committee independent of the investigators, their employing organizations, funders and sponsors will provide overall supervision of the trial. They will monitor progress and advise on scientific credibility. A data monitoring committee will assess the progress of the trial. Safety monitoring and procedure manuals will be written before the commencement of the trial and distributed to relevant research staff. All adverse events will be recorded, and recurring (i.e. occurring in multiple participants more than once during the study period) adverse events will be reported to the data monitoring committee.

### Ethics

Ethical approval was granted by Yorkshire and the Humber–South Yorkshire Research Ethics Committee in October 2023 (REC reference: 23/YH/0199) and Health Research Authority and Health and Care Research Wales (IRAS project ID: 328528).

### Dissemination

Results will be disseminated to key stakeholders and, more broadly, in several ways. These include: open‐access peer‐reviewed journal articles; presentation at key scientific meetings; feedback to trial participants via email or text message, which will include a link to blog posts regarding study results; and press releases. The data will be available on request to the principal investigators of the study.

## DISCUSSION

ESCAPE will be the first large‐scale RCT of e‐cigarette starter kits offered to adults who smoke with mental illness treated in the community in the UK. The trial is designed to provide insights into effective strategies for addressing tobacco use in this population, who face greater challenges compared with the general population owing to high dependence and limited access to support, relevant to researchers, policymakers and clinicians alike. If the intervention is shown to be both effective and cost‐effective, this will extend the evidence base for e‐cigarettes as a viable smoking cessation tool to adults who smoke with mental illness, with the potential to alleviate the substantial burden of tobacco‐related mortality and disease prevalent within this population and reduce associated health inequalities.

This is an important study that includes the use of biochemical validation of smoking abstinence, enhancing the reliability of the findings. However, there are some limitations that should be noted. First, the study is conducted in the UK, where the population with mental illness may differ from elsewhere, both in terms of healthcare access and specifically smoking cessation support, which is free to access in the UK. Additionally, this study may have limited generalizability to other countries, especially those that do not provide free smoking cessation services as part of the healthcare system and those where e‐cigarettes are regulated differently to the UK. Furthermore, owing to the nature of the intervention and study design, participants, researchers and clinical staff administering the intervention cannot be blinded. We will only recruit adults who smoke with a diagnosis of mental illness treated in the community and who are willing to address their smoking behaviour, either by attempting to quit or by reducing their cigarette consumption. Additionally, we will not conduct any analyses specifically focusing on individuals with different types of mental illness.

## CONCLUSIONS

The ESCAPE trial will be the first large‐scale study in the UK to explore the effectiveness of providing an e‐cigarette starter kit as a harm reduction and smoking cessation tool for adults who smoke with mental illness treated in the community. The finding of this study will provide much needed information to strengthen the evidence base for effective intervention strategies to address tobacco use in this population and reduce associated health inequalities.

## AUTHOR CONTRIBUTIONS

**Dimitra Kale:** Methodology; project administration; writing—original draft; writing—review and editing. **Emma Beard:** Methodology; writing—review and editing. **Yan Ding:** Methodology; project administration; writing—review and editing. **Jodi Pervin:** Methodology; project administration; writing—review and editing. **Qi Wu:** Methodology; writing—review and editing. **Catherine Arundel:** Methodology; writing—review and editing. **Steve Parrott:** Conceptualization; methodology; writing—review and editing. **Paul Galdas:** Conceptualization; methodology; writing—review and editing. **Michelle Horspool:** Conceptualization; methodology; writing—review and editing. **Simon Hough:** Conceptualization; methodology; writing—review and editing. **Gregor Russell:** Conceptualization; methodology; writing—review and editing. **Suzy Ker:** Conceptualization; methodology; writing—review and editing. **Elena Ratschen:** Conceptualization; funding acquisition; methodology; supervision; writing—review and editing. **Lion Shahab:** Conceptualization, funding acquisition; methodology; supervision; writing—review and editing.

## DECLARATION OF INTERESTS

L.S. has received honoraria for talks, unrestricted research grants and travel expenses to attend meetings and workshops from manufacturers of smoking cessation medications (Pfizer and Johnson & Johnson) and has acted as paid reviewer for grant‐awarding bodies and as a paid consultant for healthcare companies. All authors declare no financial links with tobacco companies, e‐cigarette manufacturers or their representatives.

## CLINICAL TRIAL REGISTRATION

ISRCTN17691451.

## Supporting information


**Data S1.** Supplementary Information.

## Data Availability

When the trial is completed the data will be kept in a repository for 10 years and will be available upon request.
